# Letter to the Editor

**Published:** 1912-06

**Authors:** 

**Affiliations:** Peoria, Ill.; Peoria, Ill.


### Letter to the Editor.

PEORIA, ILL., May 9, 1912.

*F. E. Daniel, M. D., Austin, Texas.*

MY DEAR DOCTOR: "The Castration of Sexual Perverts" in your April number was worth many times the subscription price of the "Red Back," as was "One Bane of Prudery" and "Pulmonary Consumption Cured by Persistent Walking, and Without Medicine," in May number. We do not speak of these because it is exceptional to find something good in the magazine, for there is *always* something good in it. We have enjoyed very much "The

Strange Case of Dr. Bruno," and we are very glad that we have become acquainted with you. Such things have never ending influences for good.

Sincerely and fraternally,

DRS. PARKER & PARKER,

